# Duchenne muscular dystrophy trajectory in R-DMDdel52 preclinical rat model identifies COMP as biomarker of fibrosis

**DOI:** 10.1186/s40478-022-01355-2

**Published:** 2022-04-25

**Authors:** Valentina Taglietti, Kaouthar Kefi, Iwona Bronisz-Budzyńska, Busra Mirciloglu, Mathilde Rodrigues, Nastasia Cardone, Fanny Coulpier, Baptiste Periou, Christel Gentil, Melissa Goddard, François-Jérôme Authier, France Pietri-Rouxel, Edoardo Malfatti, Peggy Lafuste, Laurent Tiret, Frederic Relaix

**Affiliations:** 1 Univ Paris-Est Créteil, INSERM, U955 IMRB, “Biology of the Neuromuscular System” Team, 94010 Créteil, France; 2 École Nationale Vétérinaire d’Alfort, IMRB, 94700 Maisons-Alfort, France; 3 AP-HP, Hôpital Mondor, FHU SENEC, Service de Pathologie, 94010 Créteil, France; 4 Sorbonne Université, INSERM, UMRS974, Center for Research in Myology, 75013 Paris, France; 5 EFS, IMRB, 94010 Créteil, France

**Keywords:** Duchenne muscular dystrophy, Skeletal muscle, Preclinical modelling, Translational medicine, Long QT, scRNAseq

## Abstract

Duchenne muscular dystrophy (DMD) is a fatal muscle-wasting disorder caused by mutations in the Dystrophin gene and for which there is currently no cure. To bridge the gap between preclinical and therapeutic evaluation studies, we have generated a rat model for DMD that carries an exon 52 deletion (R-DMDdel52) causing a complete lack of dystrophin protein. Here we show that R-DMDdel52 animals recapitulated human DMD pathophysiological trajectory more faithfully than the mdx mouse model. We report that R-DMDdel52 rats displayed progressive and severe skeletal muscle loss associated with fibrotic deposition, fat infiltration and fibre type switch. Early fibrosis was also apparent in the cardiac muscle. These histological modifications led to severe muscle, respiratory and cardiac functional impairments leading to premature death around 1 year. Moreover, DMD muscle exhibited systemic inflammation with a mixed M1/M2 phenotype. A comparative single cell RNAseq analysis of the diaphragm muscle was performed, revealing cellular populations alteration and molecular modifications in all muscle cell types. We show that DMD fibroadipogenic progenitors produced elevated levels of cartilage oligomeric matrix protein, a glycoprotein responsible for modulating homeostasis of extracellular matrix, and whose increased concentration correlated with muscle fibrosis both in R-DMDdel52 rats and human patients. Fibrosis is a component of tissue remodelling impacting the whole musculature of DMD patients, at the tissue level but most importantly at the functional level. We therefore propose that this specific biomarker can optimize the prognostic monitoring of functional improvement of patients included in clinical trials.

**Supplementary Information:**

The online version contains supplementary material available at 10.1186/s40478-022-01355-2.

## Introduction

Duchenne muscular dystrophy (DMD) is a progressive severe disease affecting skeletal muscles. The major symptoms are muscles wasting and weakness associated with cardiac and respiratory defects, ultimately leading to the death of the patients within the third decade of life. DMD is a genetic disorder caused by mutations in the *dystrophin* gene (*DMD*) [43]. Several thousands of mutations have been identified, and the majority of *DMD* mutations are clustered in hot spots with deletions occurring mainly in the area spanning exon 45 to 55 [5]. DYSTROPHIN is a myofibre structural protein, part of a protein complex that connects the cytoskeleton to the extracellular matrix, thus strengthening muscle fibres and protecting them from contraction-induced damage [19]. In addition, the dystrophin complex has also a signalling transduction role, by functioning as an anchor for different signalling molecules [11].

Although mouse models exhibit only a mild DMD pathological phenotype and partially recapitulate the severity of the DMD human disease, most early preclinical works were conducted in *dystrophin*-deficient mice. To bridge the translational gap between mice and humans, larger animal models of DMD have been identified or generated, including pig, dog and rhesus monkey models [35, 58]. More recently, DMD rat models have been genetically generated targeting the genomic region that spans exons 3-26 [29, 36, 46]. Surprisingly, none of these DMD rat models carry mutations in the major hot spot area of exons 45-55. Because the currently available rat models for DMD cover a limited mutational pattern, their use in translational research is limited, especially for the preclinical evaluation of therapeutic approaches that specifically target the mutation (e.g., exon-skipping antisense oligonucleotides). We thus generated a new DMD rat model with a deletion mutation in the exon 52 of the rat *Dmd* gene, and named this line R-DMDdel52. Unlike the mdx mouse model that does not exhibit severe clinical symptoms or life span reduction [8, 9], R-DMDdel52 rats display premature lethality between 10 and 14 months of age, following a process of severe dystrophic remodelling of the entire striated musculature, including the heart and diaphragm.

In this report, we provide a comprehensive set of data on the full characterisation of R-DMDdel52 rats over their lifetime, including clinical, histological, functional, and molecular observations collected during this comprehensive longitudinal analysis. We show that R-DMDdel52 rats exhibited most of the major clinical signs observed in DMD patients, recapitulating the exacerbation of the pathology with the age progression. This included progressive muscle fibrosis and inflammation, as well as replacement of muscles tissue by fat. Moreover, R-DMDdel52 rats displayed severely reduced skeletal muscle force and cardio-respiratory disease progression. Additionally, to decipher the contribution of each cell type to the molecular mechanisms of DMD pathogenesis of one of the most affected and survival-critical muscle for patient, the diaphragm, we performed a transcriptomic analysis using single-cell RNAseq (scRNAseq). We identified broad muscle cellular and molecular modifications and a new pathogenic marker of fibrosis, which is one of the major histological hallmarks of DMD, accompanying deleterious clinical progression. Indeed, we found that activated fibroadipogenic progenitors (FAPs) produced increased levels of *Comp/Thrombospondin5* transcripts, leading to elevated COMP deposition in fibrotic areas of DMD muscles as well as elevated COMP levels in the bloodstream. Cartilage oligomeric matrix protein (COMP) is a multidomain calcium binding protein characterized in fibrotic tissues of tendons, lung, skin and liver [4, 16, 24, 59], where it plays a role in extracellular matrix remodelling and in maintaining the integrity of collagen network [2, 22, 57]. In different pathological conditions COMP levels are associated with degrees of fibrosis and has been proposed as prognostic indicator for disease severity and treatment efficacy [25, 63, 66]. Moreover, endomysial fibrosis has been shown as the major muscle modification correlated with motor outcome in DMD patients [12]. Here, we identify COMP as a relevant and significant biomarker of DMD in R-DMDdel52 rats and human samples.

## Material and methods

### Rat line

The R-DMDdel52 rat model was generated by producing a 188 bp -deletion with a premature stop codon generation into the exon 52 of the Dystrophin (*Dmd*) gene. Rat exon 52 sequence (ENSRNOE00000518152) were selected and BWA algorithm was used to identify guide RNA sequences (sgRNAs) for CRISPR mediated genome editing. Exon 52 was deleted using a two sgRNA pairs approach. The sgRNA sequences are: 5′sgRNAs: ATATGAATCTGCAGTTTGCA (gR61) and CCTTTATGCTGCATAAAAGG (gR69) and 3′sgRNAs: ACCGAAGTAAGTCTCTTAAT (gR84) and TTGTGTTTTTCGTGACGGTA (gR93). The sgRNA number refers to the MIT specificity score (http://crispor.tefor.net/crispor.py). Two hundred and forty-six fertilized oocytes from the Sprague Dawley strain (RjHan:SD provided by Janvier Labs, France) were microinjected with a mix of the four sgRNAs (12 ng/µl of each) and spCas9 mRNA (25 g/µl). F0 animals were screened by genotyping by junction PCR. Genotyping is performed on DNA extracted from tail or ear biopsies. PCR mix was prepared with Dream Taq 10X buffer (ThermoFisher Scientific, Cat#B65), 0.25 mM dNTP (10 mM), 0.3 µM of forward primer (5′-CTAACGCATTTAAAATATGCTGTCA-3’), 0.3 µM reverse primer (5′-GTTGGCTTAGCTCAACAACCAAGAT-3′), 0.03 U/µl DreamTaq and around 150 ng of DNA. The PCR was performed using the Thermal Cycler 2720 with the following thermic program: initial denaturation at 95 °C for 5 min, 40 cycles with denaturation step at 95 °C for 10 s, annealing at 60 °C for 30 s and extension at 72 °C for 45 s, and the final extension at 72 °C for 5 min. Deletion of the exon 52 was detected on a 2% agarose gel.

Rats were housed in a pathogen-free facility with 12-h light and 12-h dark cycles in accordance with European Directive 2010/63/EU. Only male rats were used for the experiments. Both wild type (WT) and DMD rats were born from the same litter. All the procedures including animal handling were validated by the ethic committee of the French ministry (APAFIS#25606-202005311746599).

### Age of death

On average, the general condition of the DMD rats started to deteriorate from the age of 6 months, and we helped the weakest animals to feed by putting the food pellets on the floor. When this help was no longer sufficient and the animals were losing weight excessively with an expression of apathy, we considered that they had reached the decided humane limit point and euthanised them.

### Sample collection

All rats were anaesthetized and intraperitoneally injected with 0.1 ml/100 g of undiluted pentobarbital and then killed by cardiac puncture. Muscles (heart, diaphragm, and *tibialis anterior*) were dissected and collected for histology. For each of them, one end was placed in the gum (tragacanth gum, Sigma Aldrich) on a flat piece of cork so that the long axis is perpendicular to the cork and a major part of the muscle extends above the gum. All muscles were dipped in a cup of isopentane maintained at -140 °C by liquid nitrogen for 1 min then placed on dry ice before their transport in a -80 °C freezer until processed. Transversal muscle cuts of 7 µm of thickness were obtained using a cryostat (Leica) and used for histological analysis.

### Haematoxylin and Eosin

The samples were stained with 0.1% Mayers hematoxylin (Sigma Aldrich) for 10 min and then dipped in 0.5% eosin (Sigma Aldrich). The sections were washed in distilled water and then rinsed in 50%, 70%, 95% and then 100% ethanol before being incubated in xylene and mounted in Eurokitt.

### Picro Sirius red

Frozen tissue sections were rinsed in distilled water before the incubation in Picro Sirius red solution (Sigma Aldrich) for 25 min. Then, sections were washed in distilled water and dehydrated in 100% ethanol for 30 s before the mounting in Eurokitt.

### Immunohistochemistry

The samples were fixed in PFA 4% (Invitrogen) and permeabilized for 12 min with 0.05% Triton (Sigma Aldrich) in PBS 1X before the blocking in 10% BSA (Bovine Serum Albumin) for blocking for 45 min at room temperature (RT). Then the slides were incubated with primary antibodies: Laminin (Sigma Aldrich L9393, RRID:AB_477163), Dystrophin (Leica, NCL-DYS1, RRID:AB_442080), β-dystroglycan (Leica, B-DG, RRID:AB_442043), PDGFRα (Invitrogen, PA5-16571, RRID:AB_10981626), CD45 (BD Biosciences, 554875, RRID:AB_395568), Isolectin B4 (ThermoFisher, I21412, RRID:AB_2314664), M-cadherin (RD system biotech, AF4096, RRID:AB_10641849), CD68 (Abcam, ab31630, RRID:AB_1141557), CD206 (Abcam, ab125028, RRID:AB_10973022), COMP (Novus biologicals, NBP2-92758, RRID:AB_577733), MHC-IIa (DSHB, SC71, RRID:AB_2147165), MHC-IIb (DSHB, BFF3, RRID:AB_2266724), MHC-I (DSHB, BAD5, RRID:AB_2235587) and conjugated Laminin 647 (Novus biologicals, NB-144AF647, RRID:AB_2891039). All antibodies were diluted in 0.1% BSA and incubated overnight (o/n) at 4 °C. The slides were then exposed to Alexa Fluor secondary antibodies for 45 min at RT. After rinsing, nuclei were stained with Hoechst (Sigma-Aldrich) followed by mounting slides with Fluoromount-G Mounting Medium (Invitrogen). Photos were acquired using a fluorescent microscope (ZEISS Axio Imager 2 and LSM800 confocal).

### Western blot

Cryosections of soleus and diaphragms of 16-week-old WT and R-DMDdel52 rat were homogenized in 50 mM Tris–HCl, pH 7.4, 100 mM NaCl, 0,5% NP40 and Halt Protease inhibitor cocktail (Pierce). Protein extracted were centrifugated and denatured in Laemmli buffer for 30 min at RT. Proteins were quantified by Bradford assay (Pierce) and separated by electrophoresis (Nu-PAGE 4–12% Bis–Tris gel; Life Technologies) before being transferred to nitrocellulose membranes (GE Healthcare). Next, membranes were blocked with 5% milk for 1 h at RT and primary antibodies (dystrophin, Leica Dys2 RRID:AB_442081; and b-tubulin, Sigma RRID:AB_477576) were applied for o/n incubation at 4 °C. Next day, following washes, secondary antibodies conjugated with horseradish peroxidase were added and membranes were incubated for 1 h at RT. Following the 10 min incubation with substrate, images were acquired using Chemidoc MP (Biorad).

### RNA extraction and RT-qPCR

Quadriceps muscle samples were lyzed in Trizol (ThermoFisher) and chloroform (Sigma Aldrich) was added to recover the aqueous phase enriched of RNA. After precipitation in isopropanol, washes in 75% ethanol, the RNA pellets were air-dried and resuspended in RNA-free water. 1 μg of RNA was reverse transcribed (Transcriptase inverse SuperScript, Invitrogen) following the manufacturer’s instructions. cDNA was diluted 10 times and 2 μL was used per 10 μL of qPCR reaction. Each qPCR reaction contained 100 nM of primers and 5 μL of SYBR Green Mix (4472908, Thermo Fisher). The sequences of primers are the following: *Tgfb1* (For: AGAGAAGAACTGCTGTGTACGG, Rev: AAGTTGGCATGGTAGCCCTT, *Il6* (For: GACTTCCAGCCAGTTGCCTT, Rev: AAGTCTCCTCTCCGGACTTGT), *Ccl2* (For: GTTAATGCCCCACTCACCTG, Rev: AGTTCTCCAGCCGACTCATTG), *Comp* (For: CCGTCGCGCTCCAAAATAC, Rev: AACACTTCTGCCCCGACG), *Actb* (For: TGTCACCAACTGGGACGATA, Rev: GGGGTGTTGAAGGTCTCAAA). Fluorescence was quantitated using the Applied Biosystems, StepOne Plus Real-Time PCR (Thermo Fisher Scientific). *Actb* was used as internal housekeeping gene to normalize the data.

### scRNAseq

Bulk scRNAseq was performed on diaphragm tissues isolated from WT and R-DMDdel52 rats aged 12 months after enzymatic and mechanic digestions at 37 °C in agitation with 0.5 U/ml Collagenase A (Roche) and 3 U/ml Dispase II (Roche). Cell resuspensions were filtered, and vital cells were isolated by fluorescence-activated cell sorting (FACS) as DAPI-negative. Gel beads emulsions were obtained using Chromium Single Cell 3’ kit and clean-up cDNAs were used to prepare next-generation sequencing libraries. Sequencing was performed using Nextseq550 of Illumina. Data were analysed using Cell Ranger software and visualized via Loupe Cell Brower.

### Grip test

Forelimb grip strength was assessed using a grip strength meter (BIO-GS3, Bioseb). Animals were subjected to grasp the grid with their forelimbs, then gentle pulled back up to grip was released. The measurements were repeated 5 times with at least 10 min of rest on a flat surface between each. Maximum value was taken as an index of the strength. To correct for individual variation in strength due to growth variation, values were then normalized on the average tibial length per group, divided by the tibial length of each individual rat.

### Treadmill

Locomotion was assessed using a treadmill (LE87 10RTS, Pan lab Harvard apparatus). Belt slope was set at 5 degrees inclination and electrical shock intensity of the resting pad was set at 0.2 mA. Rats were pre-acclimated to the treadmill for 2 weeks before performing the final test. During the first day, rats were subjected to stay on the treadmill for 10 min and then rats were walking for 10 min every day at a constant speed of 5 cm/s. During the second week of training, the rats were run every two days with increasing speed and rest for two days before the final test. For test, rats ran with a start speed of 5 cm/s with acceleration of 2 cm/s every 2 min. The assay was finished when the animal stopped for 3 times in a row on the rest pad.

### Plethysmography

Respiratory functions were measured by whole body plethysmograph (Emka Technologies) on unanaesthetised rats. Individual rats were placed in a calibrated cylindrical chamber at RT. Following 5 min of acclimatization, data were recorded for 15 min and then analysed with IOX2 software.

### Electrocardiography (ECG) analyses

Electrocardiograms were performed on unsedated and unanaesthetised vigile rats using non-invasive telemetry system with two electrode cables worn in a jacket (rodentPACK, Emka Technologies). After a period of habituation of the animals to the electrodes, data was acquired during 10 to 20 min, corresponding to ~ 6000 heart revolutions. Data were further analysed by the "Averaged beats analysis" plugin of the ECGavg software (v1.0.1.4) that we codeveloped with EMKA. The software produced a graph of heart rate trends over time. For each rat, we selected the lowest heart rate value, considering that this corresponded to a resting cardiac activity devoid of the confounding impact of the hyperactivated sympathetic nervous system during the acute stress response. To this baseline value we added 10% and analysed all cardiac revolutions between the two values (in the above example, between 400 and 440 bpm). A total number of 540 to 1934 revolutions were analysed. The ECGavg software then produced an average electrocardiogram which we annotated and used as a basis for quantifying the intervals and segments (expressed in milliseconds, ms) which we then analysed using GraphPad (version 9.3.1, Prism). By analogy with the QTc interval that was difficult to obtain for some rats because of the fluctuating isoelectric line, the heart rate corrected QTpc value corresponded to the interval between the beginning of Q and the peak of the T wave, corrected with the Bazett's formula normalized to the average rat RR (QTpc = QTp(eak) / (RR / f)1/2, f = 150 ms) [34].

### Rat COMP/THROMBOSPONDIN-5 ELISA Kit

Concentrations of circulating COMP were quantified using a commercial enzyme-linked immunosorbent assay (ELISA) kit (Novus biologicals, NBP2-82,142). Recombinant COMP standards ranging from 1.56–100 ng/ml, and serum samples were pipetted into wells precoated with specific antibody to COMP. After incubation, detection antibody solution was dispensed, and absorbance was read after treatment of the samples with the substrate solution. The optical density is clearly proportionate to the quantity of COMP in the samples and the COMP concentration were extrapolated from the standard curve reference.

### Human muscle samples

DMD was confirmed in all patients by genetic analyses and absence of dystrophin on immunoblots and cryosections. The possibility to use material for research purposes was obtained from parents or legal representatives who gave their written informed consent for the participation of the children to the study. Ethical issues were in accordance with current French legislation and hospital research ethics committee (Approval #12-009 at CPP Ile-de-France IX). For each DMD patient, 7 μm cryostat cross-sections from deltoid muscle samples were processed. Immunofluorescence experiments were performed following the same procedure for the rat sections and described above.

## Results

### Premature death, absence of dystrophin expression and muscle atrophy in R-DMDdel52 rats

**Fig. 1:**
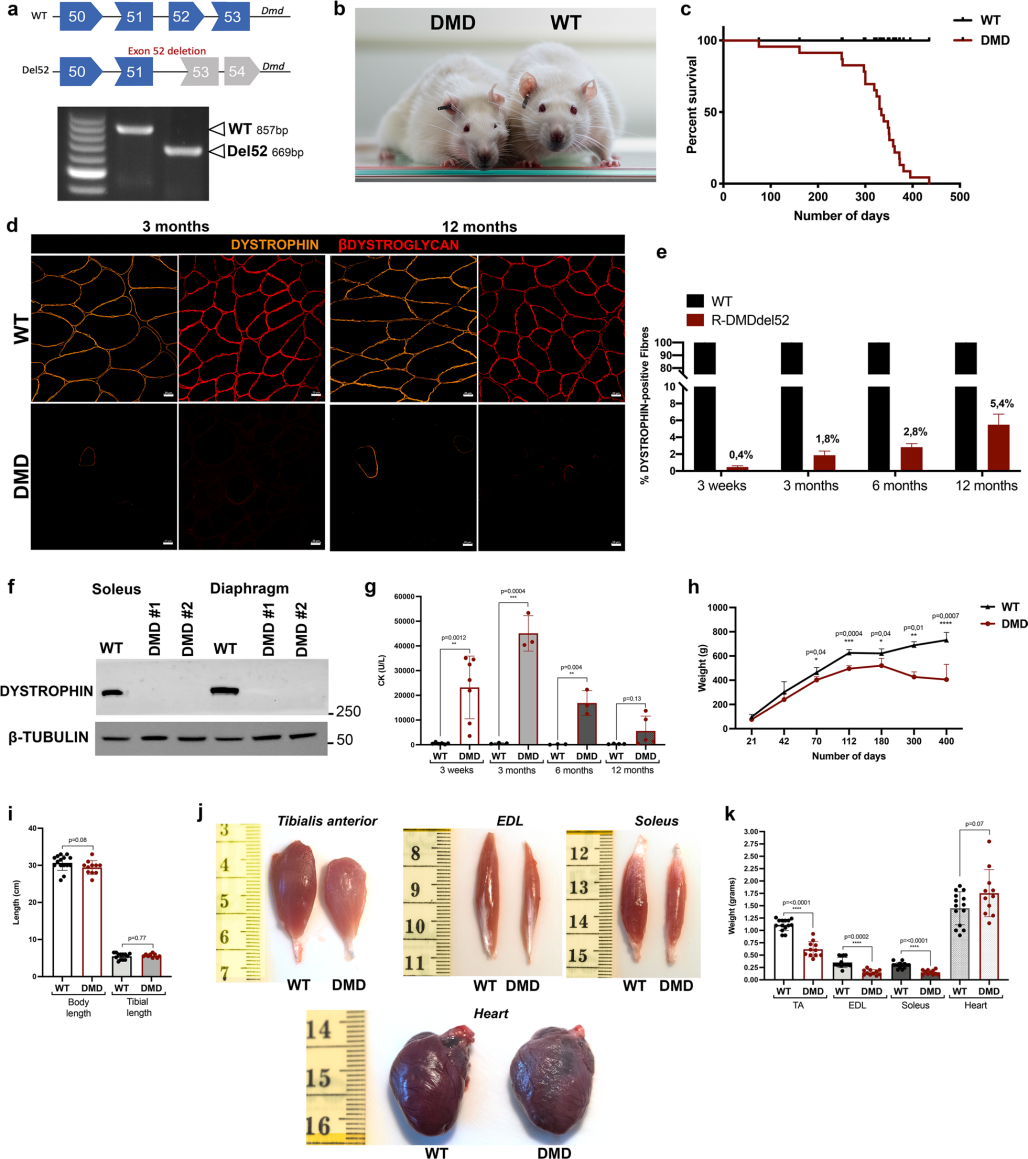
Premature death, absence of dystrophin expression and muscle atrophy in R-DMDdel52 rats. **a** Scheme of CRISPR-mediated gene deletion in rats and genotyping data from WT and R-DMDdel52 animals. Position of WT and deletion (del52) bands is indicated. **b** Pictures of R-DMDdel52 rats (left) and a WT littermate (right) aged 12 months, showing a marked facial muscle atrophy that modifies the appearance of the animal. **c** Kaplan Meier Curve for the frequency of WT (black curve) and R-DMDdel52 (red curve) rat survival. **d** Representative immunofluorescence for DYSTROPHIN (orange) and ßDYSTROGLYCAN (red) in *tibialis anterior* sections of 3-month-old (left panels) and 12-month-old (right panels) WT and R-DMDdel52 rats. Scale bar 20 μm. **e** Quantification of the percentage of dystrophin-positive fibres in WT and R-DMDdel52 TA at 3 weeks, 3, 6, 12 months of age. **f** Western blot analysis of WT and R-DMDdel52 proteins extracted from soleus and diaphragm tissues. The amount of loaded proteins was normalized on β-TUBULIN levels. **g** CK levels of WT and R-DMDdel52 rats aged 3 weeks, 3 months, 6 months and 12 months. **h** Weight curve showing that R-DMDdel52 rats exhibited a progressive loss of weight compared to their healthy littermates (WT). i) Graph showing the body and tibial length in centimetres of R-DMDdel52 and WT rats. j) Pictures of R-DMDdel52 muscles at 12 months of age: *tibialis anterior*, extensor digitorum longus, soleus, and heart. k) Graph showing the weight in grams of different skeletal muscles from R-DMDdel52 rats and WT aged 12 months

We generated a DMD rat model by injecting the Cas9/sgRNA constructs in Sprague Dawley zygotes, to delete exon 52 of the *Dmd* gene (R-DMDdel52). This 188 bp-deletion is predicted to disrupt the coding frame and produce a loss-of-function allele. Exon 52 deletion confirmed by PCR amplification with primers spanning the deletion site was used to genotype the animals (Fig. 1a). The rats were observed daily and showed increasing locomotor weakness and muscle atrophy, particularly visible in the face starting from 6 months of age (Fig. 1b). Most of the animals experienced a worsening of their body condition between 10 and 13 months of age, and not a single R-DMDdel52 rat survived beyond 14 months of age (Fig. 1c). We analyzed dystrophin protein expression on transverse *tibialis anterior* (TA) sections by immunofluorescence. In parallel, we also performed an immunostaining of β-dystroglycan, as component of the dystrophin-associated protein complex. Both dystrophin and β-dystroglycan proteins were nearly absent in R-DMDdel52 muscles, which however displayed rare revertant fibres (Fig. 1d). We quantified the percentage of revertant fibres from 3 weeks until 12 months, the oldest age beyond which the number of surviving animals was insufficient to obtain statistically analysable data. During the lifespan of R-DMDdel52 rats, we observed an increased percentage of revertant fibres by immunostaining (Fig. 1d, e). Despite these, western blot analysis could not detect any dystrophin protein in soleus and diaphragm of R-DMDdel52 rats, compared to their wild-type (WT) counterparts (Fig. 1f). Next, we measured the serum levels of creatinine kinase (CK), a protein biomarker of fibre damage (Fig. 1g). CK levels (U/l) were elevated in serum of R-DMDdel52 rats at early time points and then they sharply decreased with age as the muscle mass was substituted by fibrotic and fat tissue. We also recorded rat body weight from 21 days of age until the death of the animals. With this panel of rats, we observed no significant differences in body weight between genotypes between 21 and 42 days; however, starting from 2 months of age, the body weight was significantly decreased in R-DMDdel52 rats compared to controls, with a marked drop after 6 months of age (Fig. 1h, Table 1). Of note, there was no significant difference in body and tibial length in the R-DMDdel52 cohort compared to WT at 12 months of age (Fig. 1i), emphasizing, that the reduced body weight was not due to a growth defect but rather reflected the severe muscle atrophy visually observed in dystrophic rats. To confirm this at the muscle level, we sampled muscles in R-DMDdel52 rats aged 12 months. *Tibialis anterior* (TA), *extensor digitorum longus* (EDL) and soleus of DMD rats appeared smaller than those of the WT littermates (Fig. 1j) and we quantified a significant reduction of their weight (Fig. 1k, Table 1). Altogether, these data confirmed that R-DMD rats suffered from a severe progressive skeletal muscle atrophy, sufficient to cause a substantial decrease in their body mass by the age of 2 months. Conversely, the hearts of R-DMDdel52 rats aged 12 months appeared slightly bigger than those from WT (Fig. 1j) with a tendency towards an increased weight at this age (Fig. 1k).

**Table 1:** Body weights, body mass index, body length and muscle weights

Parameter	3 weeks		6 months		12 months	
	WT	DMD	WT	DMD	WT	DMD
Body weight (g)	96.9 ± 21.4	76.0 ± 3.4	621.3 ± 38.4	547.2 ± 60.0*	731.1 ± 63.3	337.7 ± 88.9***
Body Mass Index (g/cm^2^)	0.39 ± 0.03	0.34 ± 0.03	0.70 ± 0.06	0.63 ± 0.04	0.74 ± 0.04	0.34 ± 0.22**
Body length (cm)	15.7 ± 1.2	14.9 ± 0.6	29.1 ± 1.0	29.0 ± 1.3	30.5 ± 1.1	29.6 ± 0.7
Tibialis anterior (g)	0.16 ± 0.03	0.12 ± 0.01	1.21 ± 0.34	0.97 ± 0.22	1.15 ± 0.05	0.55 ± 0.21**
Heart (g)	0.47 ± 0.06	0.47 ± 0.05	1.77 ± 0.10	1.84 ± 0.48	1.86 ± 0.11	2.20 ± 0.45§
Soleus (g)	0.054 ± 0.03	0.035 ± 0.02	0.39 ± 0.03	0.25 ± 0.13	0.35 ± 0.10	0.15 ± 0.06*

*p* value are calculated refered to WT

*p < 0.05

**p < 0.01

***p < 0.001

^§^*p* = 0.07

### Muscle functional evaluations in R-DMDdel52 rats

**Fig. 2:**
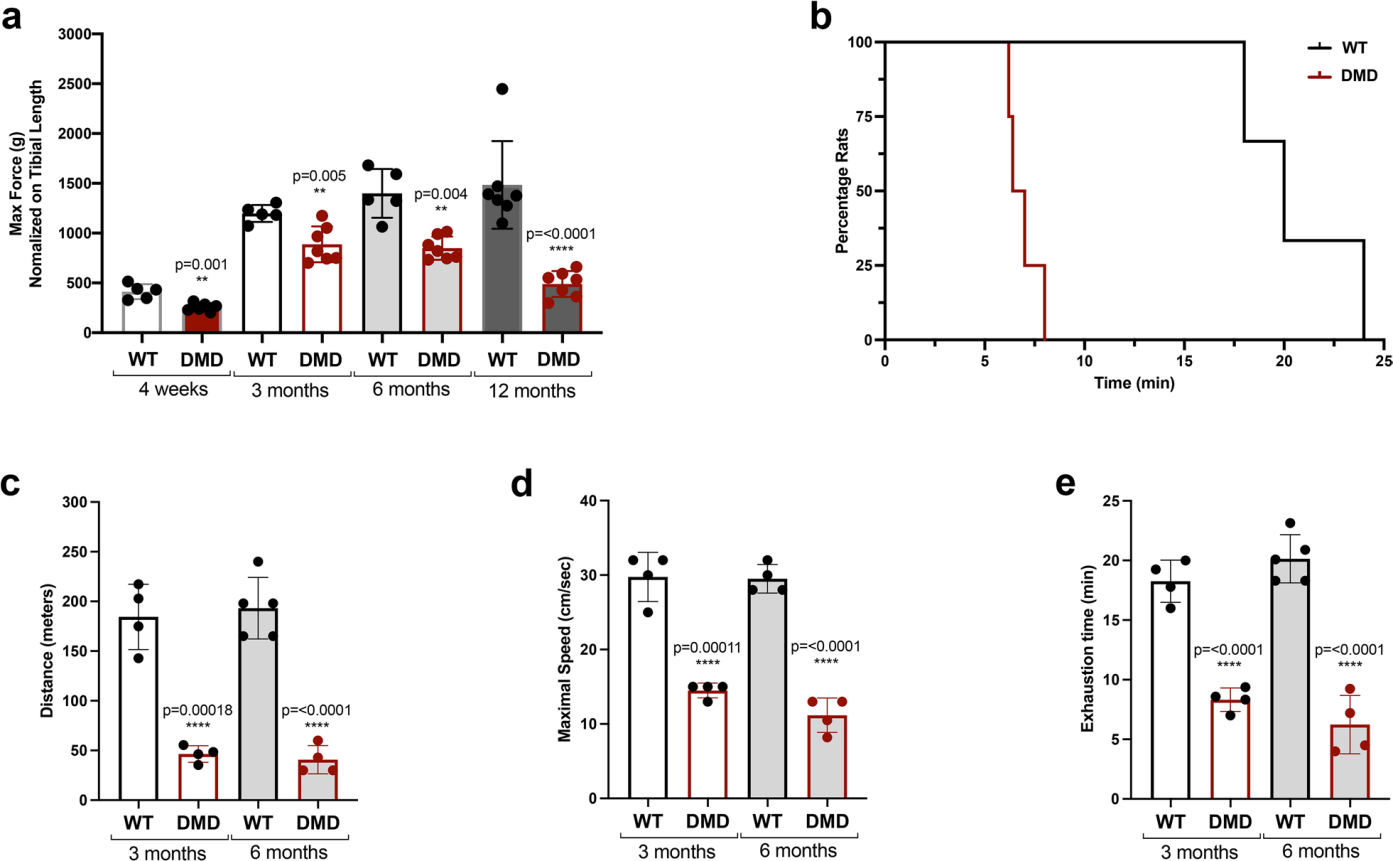
Functional impairment of R-DMDdel52 animals. **a** Maximum force applied during grip test at 3 weeks, 3, 6 and 12 months of age in WT and R-DMDdel52 rats. **b** Graph showing the percentage of exhausted rats in time during the treadmill test, performed on WT and R-DMDdel52 animals aged 6 months. **c**–**e** Maximal distance in meters (**c**), maximal speed expressed in cm/s (**d**) and exhaustion time in minutes (**e**) measured during the treadmill test performed on R-DMDdel52 and WT rats aged 3 and 6 months

To assess skeletal muscle performance in vivo, we performed both grip assay and treadmill exhaustion test. The grip test was used to measure muscular strength in WT and R-DMDdel52 rats aged 3 weeks, 3, 6 and 12 months, showing a statistically significant decrease in the maximum force applied by R-DMDdel52 forelimbs compared to WT, at all the analysed time points (Fig. 2a). In addition, we used the treadmill test to determine endurance and fatigue, with the animals running until exhaustion with a gradually increasing speed. During this exercise, R-DMDdel52 rats became exhausted significantly earlier than WT rats (Fig. 2b). Indeed, both at 3 and 6 months, R-DMDdel52 rats ran a reduced distance (Fig. 2c) at a lower speed (Fig. 2d), and during a shorter time (Fig. 2e) compared to aged-matched WT animals, confirming a severe functional impairment in their mobility.

### Diaphragm and respiratory evaluations in R-DMDdel52 rats

**Fig. 3:**
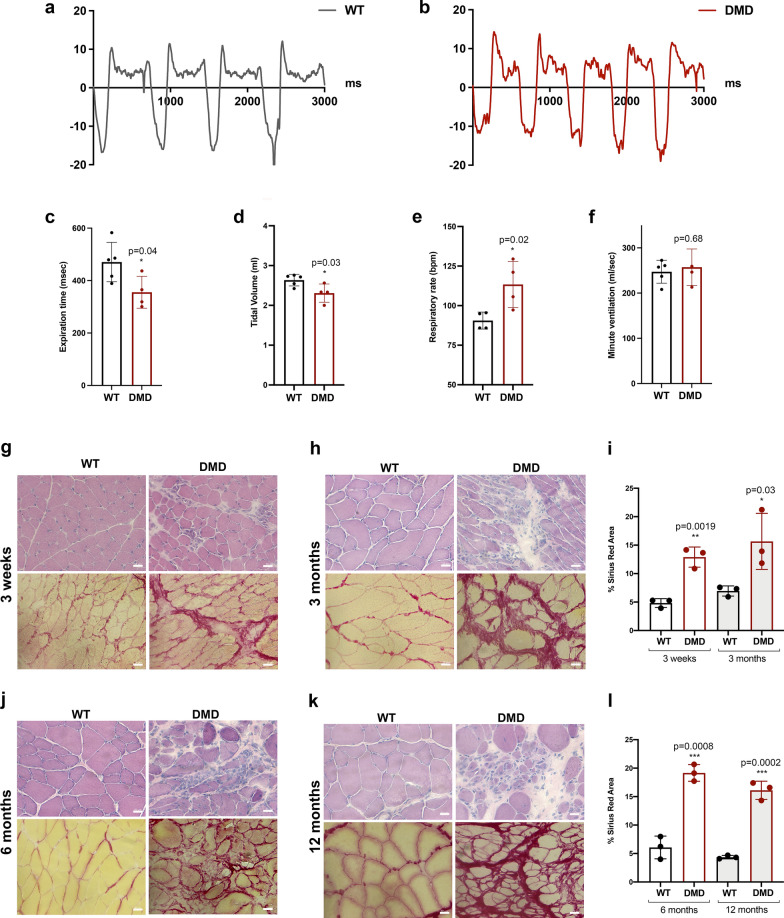
Diaphragm and respiration assessment in R-DMDdel52 rats. **a**, **b** Recording trace of respiratory flow of WT (**a**) and R-DMDdel52 rats (**b**) aged 6 months under baseline conditions. Negative flow peaks indicate depression in the box, i.e. the inspiration step for the animal, while the positive ones reflect the expiration. **c**–**f** exhalation time (**c**), tidal volume (**d**), respiratory rate (**e**) and minute ventilation (**f**) measured by whole body plethysmography in R-DMDdel52 and WT rats aged 6 months. **g**, **h** Hematoxylin and eosin (upper panels) and Picro Sirius red (lower panels) staining of diaphragm of 3-week-old (**g**) and 3-month-old (**h**) WT and R-DMDdel52 rats (scale bar 50 μm). **i** Percentage of Sirius red area in diaphragm of 3-week-old and 3-month-old WT and DMDdel52 rats. **j**, **k** Hematoxylin and eosin (upper panels) and Picro Sirius red (lower panels) staining of diaphragm at 6 months (**j**) and 12 months (**k**) of age of both WT and R-DMDdel52 rats (scale bar 50 μm). **l** Percentage of Sirius red area in diaphragm of WT and DMDdel52 rats aged 6 and 12 months

Respiratory functions were assessed by non-invasive whole-body plethysmography in R-DMDdel52 and WT rats aged 6 months. At this age, R-DMDdel52 rats showed a significant shorter exhalation time during a normal breath (Fig. 3a–c), a lower tidal volume measured at each ventilation (Fig. 3d) and a compensatory increased respiratory rate (Fig. 3e) that eventually resulted in a similar minute ventilation between WT and R-DMDdel52 (Fig. 3f). These data highlighted a severe muscular phenotype in skeletal muscle performance of R-DMDdel52 rats. In artificial living conditions characterised by limited movement in an animal cage, the lack of air exchange volume through reduced amplitude ventilation is compensated by the corrective mechanism of increased rate, which ensures sufficient haematosis.

We performed histological analysis by hematoxylin and eosin (H&E) and Picro Sirius red staining on sections of diaphragm from WT and R-DMDdel52 rats aged 3 weeks, 3, 6 and 12 months. R-DMDdel52 diaphragm were already severely affected at 3 weeks of age, showing an altered fibre morphology with invading inflammatory cells and fibrotic deposition (Fig. 3g). Fibrosis worsened with disease progression (Fig. 3h, j, k). Indeed, we quantified the extent of fibrosis by measuring the percentage of area occupied by connective tissue, marked by fluorescent Picro Sirius red, and showed statistically significant differences between WT and R-DMDdel52 diaphragm at all the time points, culminating at 6 months (Fig. 3j) when the fibrotic deposition reached its maximal levels (Fig. 3l). Next, we characterized changes in diaphragm cross-sectional area and the percentage of fibres at 3 weeks and 3, 6, 12 months in R-DMDdel52 and WT rats (Additional file 1: Fig. S1a-d). We observed a strong myofibre atrophy with decreased fibre calibres in R-DMDdel52 diaphragm at all the time points. Finally, we confirmed the severity of the DMD phenotype in a limb muscle, the TA, in terms of muscle morphology, inflammatory infiltration, myofibre heterogeneity and most importantly muscle fibrosis (Additional file 1: Fig. S1e-j).

These data further demonstrated that R-DMDdel52 rats display a severe dystrophic phenotype, with skeletal muscles being compromised and the morphological and functional level, due to the substitution of myofibres with fibrotic tissue.

### Cardiac functional and histological assessment in R-DMDdel52 rats

**Fig. 4:**
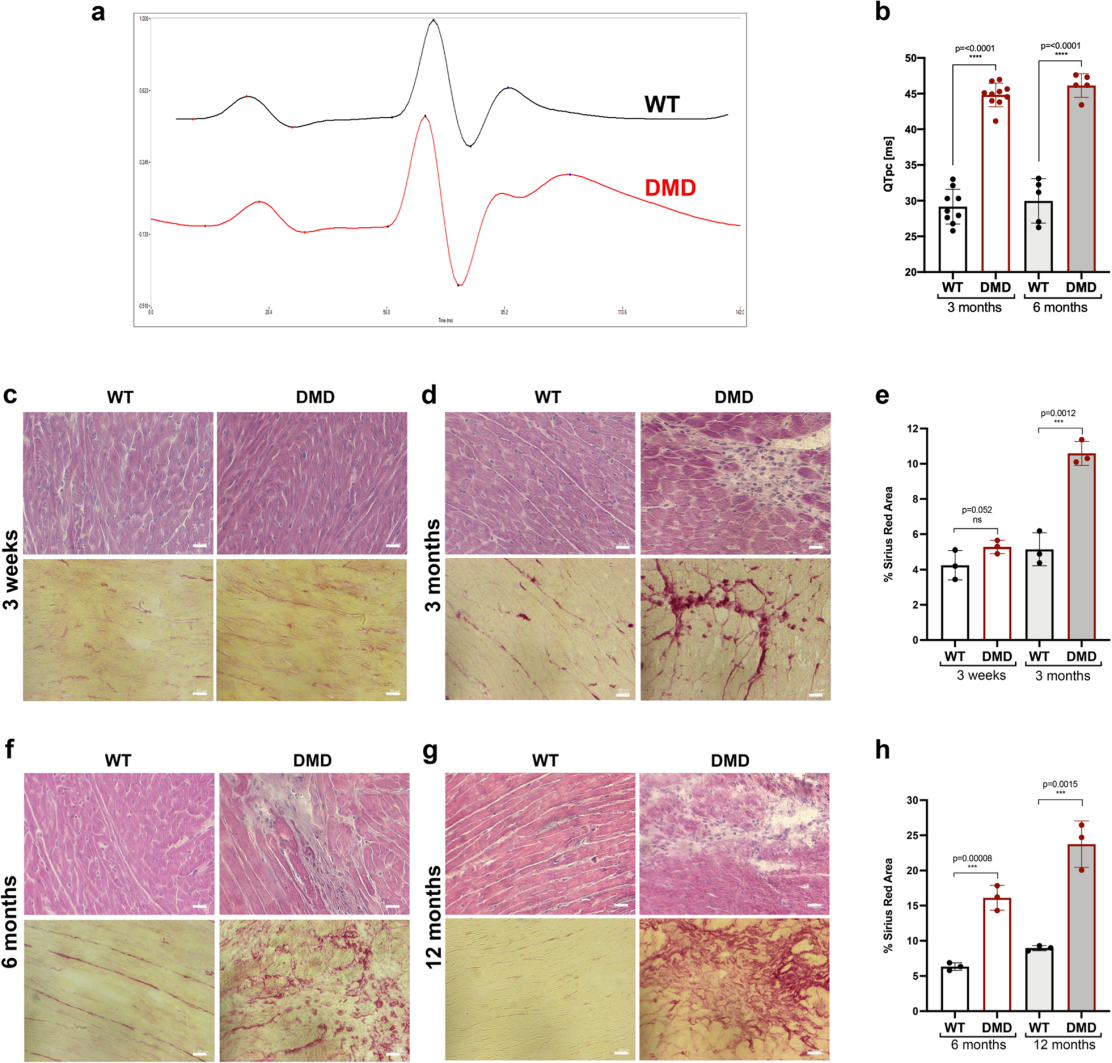
Heart functional and histological evaluation in R-DMDdel52 rats. **a** ECG signal of (black) control and (red) R-DMDdel52 rat at 6 months of age. **b** Quantification of the QTpc interval in WT and R-DMDdel52 rats at 3 and 6 months of age. **c** Hematoxylin and eosin (upper panel) and Sirius red (lower panel) staining of heart of 3-week-old WT and R-DMDdel52 rats (scale bar = 50 μm). **d** Hematoxylin and eosin (upper panel) and Sirius red (lower panel) staining of heart of WT and R-DMDdel52 rats aged 3 months (scale bar = 50 μm). **e** Quantification of fibrotic area in the hearts of WT and R-DMDdel52 rats aged 3 weeks and 3 months. **f** Hematoxylin and eosin (upper panel) and Sirius red (lower panel) staining of heart of WT and R-DMDdel52 rats aged 6 months (scale bar = 50 μm). **g** Hematoxylin and eosin (upper panel) and Sirius red (lower panel) staining of heart WT and R-DMDdel52 rats aged 12 months (scale bar = 50 μm). **h** Quantification of fibrotic area in the hearts of WT and R-DMDdel52 rats aged 6 and 12 months

Heart failure following a dystrophic process pathognomonic of the described disorder in skeletal muscle is one of the major causes of death in DMD children [48, 69]. Early electrocardiogram (ECG) abnormalities, preceding functional failure, are commonly reported in patients [41, 56, 62]. We thus characterized the cardiac phenotype of R-DMDdel52 rats and performed ECG on animals, analysed in a time window corresponding to animals at rest based on both their quiet behaviour and a similarly low heart rate (e.g. at 3 months, heart rate = 458 ± 20 s.e.m bpm in R-DMDdel52 rats and 488 ± 6 s.e.m. bpm in their WT littermates, *p* = 0.133), reflecting the absence of a stress-activated sympathetic system stimulation. Between 4 weeks and 12 months of age and whatever the genotype, all the animals analysed had a regular sinus rhythm. Remarkably, regardless of age, we observed a modified ECG pattern in all DMD rats, characterised by a bifid shape of the junction between the QRS and the T wave, the apex of which was shifted to the right (Fig. 4a). Thus, the quantification of the interval between the Q wave and the T wave peak, finely corrected for each individual by the heart rate (QTpc), was significantly increased in DMD rats compared to their WT littermates (Fig. 4b). The prolonged QTpc interval in R-DMD rats, pointed to an activation or repolarization defect (Fig. 4a), possibly induced by intramyocardial fibrosis [18]. We stained sections of hearts with hematoxylin and eosin and Picro Sirius red, sampled from R-DMDdel52 rats and their WT littermates aged 3 weeks, 3, 6 and 12 months (Fig. 4c–h). At 3 weeks of age, no clear sign of fibrotic deposition and the invading inflammatory cells routinely seen in dystrophic skeletal muscles were seen in the heart (Fig. 4c). In contrast, marked signs of cardiac muscle degeneration and fibrosis were present at 3 months of age (Fig. 4d, e), and were exacerbated at 6 and 12 months (Fig. 4f–h). We conclude from these data that the early and quantifiable ECG abnormality is highly specific to the R-DMDdel52 rats. It is most likely a consequence of the replacement of normal heart tissue by fibrosis, for which the QTpc value appears to be predictive, in addition to be highly predictive of the genotype of animals.

### Fat replacement of muscle tissue and inflammation in R-DMDdel52 muscles

**Fig. 5:**
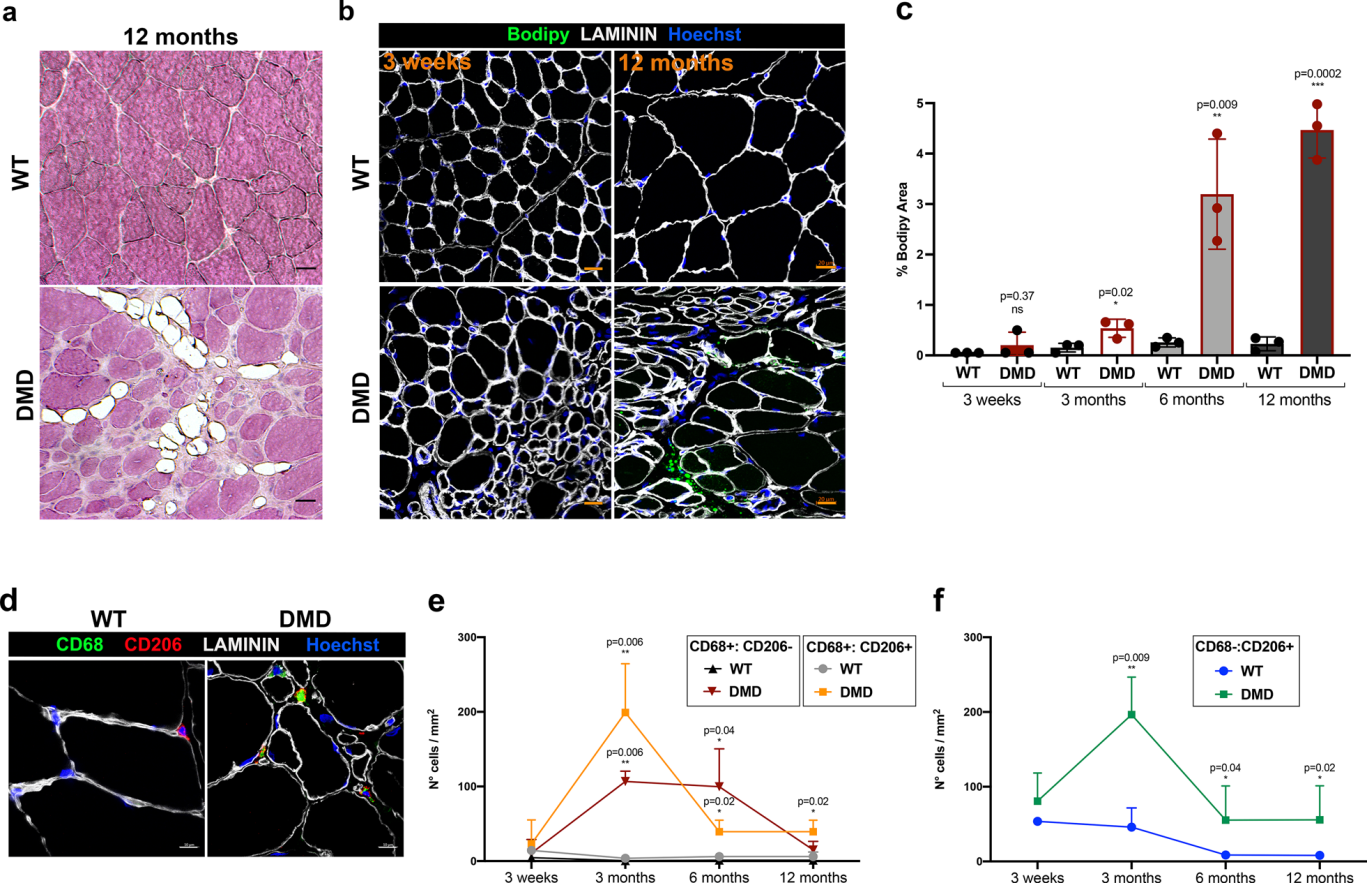
Alterations in fat deposition and inflammatory status in R-DMDdel52 diaphragms. **a** Hematoxylin and eosin staining of TA from WT and R-DMD-del52 rats aged 12 months (scale bar 20 μm). **b** Lipid droplet staining by Bodipy immunofluorescence (green) on TA of WT and R-DMDdel52 rats aged 3 weeks and 12 months (scale bar 20 μm). Laminin (white) delineates muscle fibres and nuclei are counterstained with Hoechst (blue). **c** Quantifications of b. **d** Immunofluorescence for CD68 (green), CD208 (red), and laminin (white) performed on diaphragms isolated from WT and R-DMDdel52 rats aged 6 months. Nuclei were counterstained with Hoechst (blue). Scale bar 10 μm. **e** Quantification of the number of CD68-positive or CD206-positive macrophages in 3-week-old and 3-, 6-, 12-month-old WT and R-DMDdel52 muscles. **f** Quantification of the number of CD68-negative and CD206-positive macrophages (CD206^−^:CD68^+^) in 3-week-old and 3-, 6-, 12-month-old WT and R-DMDdel52 muscles

Another hallmark of DMD is the replacement of myofibres by fat tissue. The accumulation of adipocytes within the skeletal muscle was firstly appreciated by haematoxylin and eosin, with clear fatty infiltrate adipocyte present in skeletal muscles at 12 months of age (Fig. 5a). The quantification of fat deposition was performed using staining with Bodipy, a standard lipid dye [60], on transversal sections of TA isolated from R-DMDdel52 and WT rats aged 3 weeks, 3, 6 and 12 months (Fig. 5b, c). From 3 months of age, the lipid content was elevated in intramuscular spaces of R-DMDdel52 muscles compared with WT samples. In addition, adipose infiltration increased with disease progression, reaching a peak at 12 months (Fig. 5c). Since dystrophin deficiency in striated muscles causes a degenerative process coupled with the invasion of inflammatory cells into the muscle, we decided to quantify the number of macrophages infiltrating the skeletal muscles of R-DMDdel52 animals and their WT littermates. We immunolabelled macrophages on sections of TA sampled from R-DMDdel52 and WT rats aged 3 weeks, 3, 6 and 12 months (Fig. 5d–f). From 3 months of age, R-DMDdel52 muscles showed an increased number of both M1 (CD68^+^:CD206^−^) and M2 (CD68^+^:CD206^+^) macrophages (Fig. 5e). Moreover, we noticed a higher number of cells expressing only CD206 (CD68^−^:CD206^+^) in R-DMDdel52 muscles, indicating a persistent presence of immature dendritic cells or macrophages without the expression of the cell surface marker CD68 (Fig. 5f). To better evaluate the inflammatory status of R-DMDdel52 muscles, we also performed qPCR detection of inflammatory cytokines (*Tgfb, Il6, Ccl2*) on cDNAs obtained from quadriceps of R-DMDdel52 rats and their WT littermates (Additional file 1: Fig. S2a-d). At 3 weeks, 3, 6 and 12 months of age, the expression levels of the three cytokines were significantly higher in R-DMDdel52 muscles, except for *Il6* that was found unchanged in the R-DMDdel52 quadriceps at 12 months (Additional file 1: Fig. S2d). All these data indicate that R-DMDdel52 muscles are characterized by severe and persistent chronic inflammation consistent with an exacerbation of the pathological phenotype. Finally, because it has been reported that fast-twitch glycolytic fibres (Type IIb) are preferentially affected by the disease whereas oxidative slow-twitching fibres (Type I) are mostly spared [67, 71], we characterized the fibre type distribution in the fast-twitching EDL muscle. We confirmed that in the R-DMDdel52 model, type IIb fibres were mainly affected and were progressively lost, in contrast with type I fibres that we found mostly preserved. Hence, R-DMDdel52 muscles contained a higher percentage of type I fibres at 12 months, compared to WT (Additional file 1: Fig. S2e-f).

### Comparative transcriptomic signature of single cells (scRNAseq) in the diaphragm

**Fig. 6:**
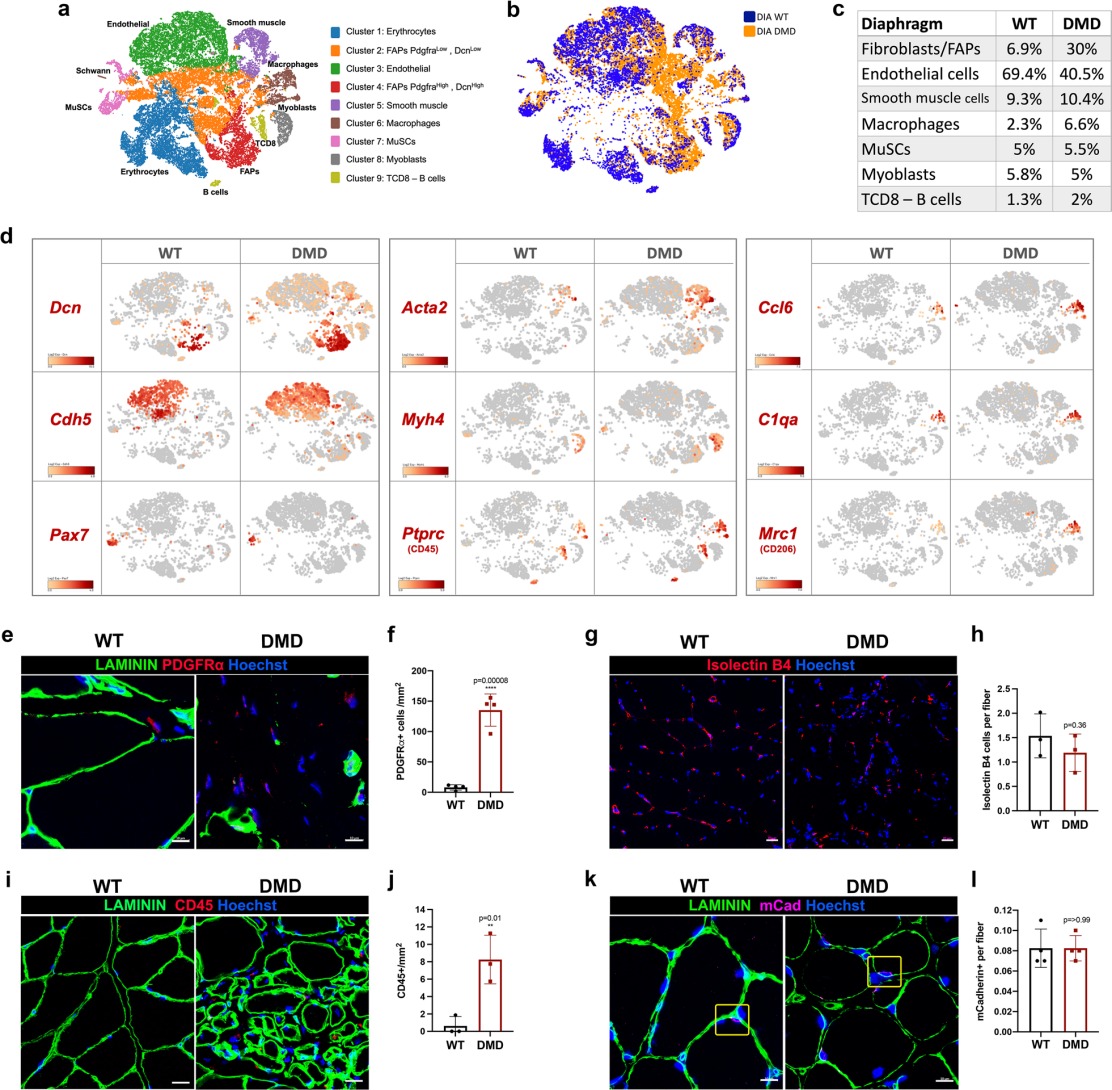
scRNAseq on 12-month-old R-DMDdel52 diaphragm compared to WT. **a** T-SNE clustering of rat muscle single-cell into nine populations. **b** Clustering overlay of WT and DMD muscle single-cells. **c** Proportion of major cell time of rat WT and DMD muscle cells. **d** Profiles of known markers in each cell population cluster (*Dcn, Cdh5, Pax7, Acta2, Myh4, Ptprc, Ccl6, C1qa, Mrc1*). **e** Representative immunofluorescence for PDGFRα (red) and LAMININ (green) in WT and DMD diaphragms at 12 months of age. Nuclei are counterstained with Hoechst (blue) (scale bar = 10 μm). **f** Quantification of the number of PDGFRα-positive cells per mm^2^ in WT and DMD diaphragms at 12 months of age. **g** Representative staining for ISOLECTIN B4 (red) and nuclei (blue) in diaphragms isolated from WT and DMD rats aged 12 months (scale bar = 20 μm). **h** Quantification of ISOLECTIN B4-positive vessels per fibre in TA of WT and DMD rats aged 12 months. **i** Representative immunofluorescence for CD45 (red) and LAMININ (green) in WT and DMD diaphragms at 12 months of age. Nuclei are counterstained with Hoechst (blue) (scale bar = 10 μm). **j** Quantifications of CD45-positive cells per mm^2^ in WT and DMD diaphragms at 12 months of age. **k** Representative immunofluorescence for mCADHERIN (purple) and LAMININ (green) in WT and DMD diaphragms at 12 months of age. Nuclei are counterstained with Hoechst (blue) (scale bar = 10 μm). **l** Quantifications of the number of mCADHERIN-positive satellite cells per fibre in diaphragms isolated from WT and DMD rats at 12 months

Using RNAseq, we assayed the transcriptomic profile of diaphragms at the single-cell resolution (scRNAseq), both in R-DMDdel52 rats aged 12 months and their WT littermates (Fig. 6a–c; Table 2). scRNA sequencing was performed on a 10X Genomics Chromium platform with unbiased clustering, and cell identity was attributed by the expression of known cell type markers [51]. A first global analysis revealed massive changes in cell populations of the diaphragm of R-DMDdel52 rats. Notably, we found a marked increase in the relative numbers of FAPs and inflammatory cells (macrophages, CD8^+^ T cells and B cells) (Fig. 6b). Differences in cell type abundancy between R-DMDdel52 and WT were confirmed by a tSNE plot based on canonical cell markers (FAPs, *Dcn*; endothelial cells, *Cdh5*; muscle stem cell, *Pax7*; smooth muscle cells, *Acta2*; myoblasts, *Myh4*; inflammatory cells, *Ptprc*) (Fig. 6d). To validate the increased number of FAPs in DMD skeletal muscles, we quantified the number of PDGFRα-positive cells on transverse sections of muscle sampled from R-DMDdel52 and WT rats at the age of 12 months, and confirmed in situ the dramatic increase in the number of PDGFRα + cells (Fig. 6e, f). Of note and in contrast with a putative decreased number of endothelial cells suggested by scRNA-seq data (Fig. 6c), we found an unchanged number of capillaries per myofibre at the same age (Fig. 6g, h), suggesting that the vascular endothelium was underrepresented in the scRNAseq analysis due to impaired digestion of muscle interstitium under dystrophic remodelling conditions. The presence of inflammatory cells in R-DMDdel52 and WT muscles was evaluated by immunostaining for CD45, a pan-leukocyte marker (Fig. 6i). The quantifications of the number of CD45-positive cells revealed a stronger presence of immune cells in 12-month-old R-DMDdel52 muscles compared to WT, confirming the results of scRNAseq (Fig. 6j). Finally, we validated that the number of satellite muscle stem cells (MuSCs, m-Cadherin +) did not vary between DMD and WT rats (Fig. 6k, l).

**Table 2:** 10X Genomics scRNAseq parameters

	Estimated number of cells	Mean reads per cell	Median genes per cell	Number of reads	Valid barcodes	Sequencing saturation
WT	2930	169,425	752	496,416 307	98%	91.3%
DMD	4812	74,686	1374	359,387 049	97.3%	88.7%

### COMP expression in the fibrotic deposition of rat and human DMD samples

**Fig. 7:**
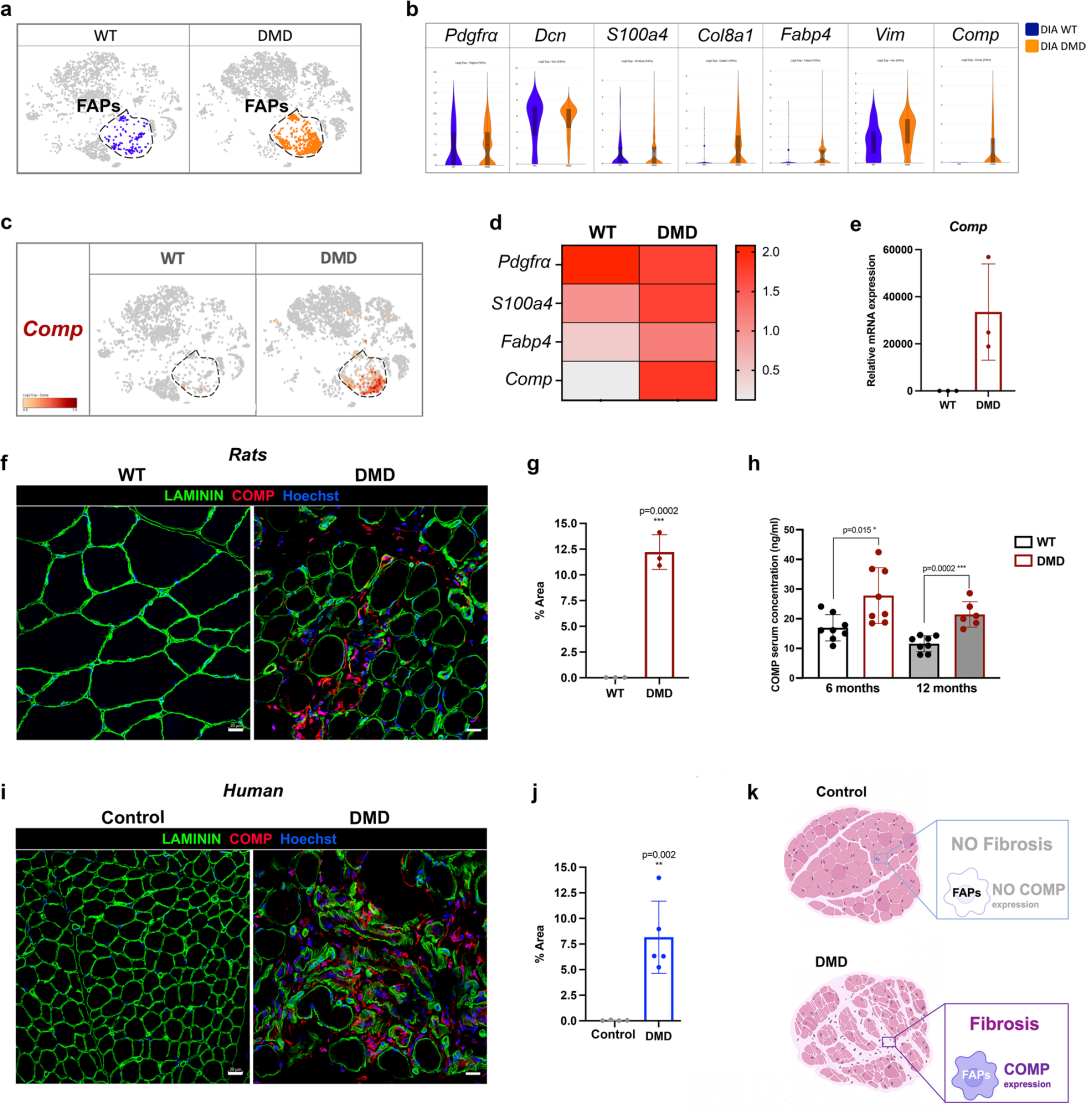
Specific Comp expression in DMD FAPs. **a** T-SNE clustering highlighting FAP population. **b** Violin plots showing the level of expression of *Pdgfrα, Dcn, S100a4, Col8a1, Fabp4, Vim and Comp* in WT and DMD diaphragms at 12 months of age. **c** T-SNE visualisation of *Comp* expression in WT and DMD FAPs. **d** Heatmap showing the relative expression of *Pdgfrα, S100a4, Fabp4* and *Comp* in WT and DMD FAPs. **e** Validation of *Comp* mRNA expression level in WT and DMD quadriceps at 12 months of age. **f** Representative immunofluorescence of COMP (red), LAMININ (green) and nuclei (blue) in 12-month-old WT and DMD rat *tibialis anterior* (scale bar = 20 μm). **g** Quantification of COMP expression area (**f**) at 12 months for WT and DMD rats. **h** Concentration of circulation COMP in sera of WT and R-DMDdel52 rats at 6 and 12 months of age. Values are expressed in ng/ml. **i** Representative immunofluorescence of COMP (red), LAMININ (green) and nuclei (blue) on control and DMD deltoid biopsies (scale bar = 20 μm). **j** Quantification the percentage of COMP-positive area on control and DMD human biopsies. **k** Graphic scheme of COMP expression in DMD fibrotic deposition

Since FAPs mediate muscle fibrosis, we further investigated their transcriptomic profile. First, we identified FAP populations in WT and R-DMDdel52 samples by the expression of PDGFRα (Fig. 7a). Then, we compared the transcriptional landscapes of WT and R-DMDdel52 FAP cell clusters showing similar level of expression of known fibroblast markers as *Pdgfra*, *Decorin* (*Dcn*), *Vimentin* (*Vim*) and *S100a4*. Overall, FAPs isolated from dystrophic samples displayed higher level of *Collagen8a1* (*Col8a1*), and *fatty acid-binding protein 4* (*Fabp4*) (Fig. 7b). Interestingly, FAPs from R-DMDdel52 muscles also expressed higher levels of *Cartilage Oligomeric Matrix Protein* (*Comp*), also known as *Thrombospondin-5*, coding for an extracellular glycoprotein mainly found in bone, cartilage tissues and in fibrotic depositions [1, 45, 61] (Fig. 7c, d). The increased expression level of *Comp* in dystrophic rat tissues was confirmed in the quadriceps by a comparative qPCR analysis performed in R-DMDdel52 and WT rats aged 12 months (Fig. 7e), followed by immunohistochemistry on TA of the same rats that confirmed elevated COMP levels, specifically within the increased endomysial molecular layer (Fig. 7f, g). We also noticed COMP accumulation in fibrosis area of heart and diaphragm of R-DMDdel52 rats at the age of 12 months (Additional file 1: Fig. S3a-c). Notably, WT samples were negative for COMP, suggesting that it is a specific marker of DMD fibrotic deposition across different muscles. To assess the potential of COMP as a circulating biomarker, we evaluated its concentration in the serum of DMD and WT rats aged 6 and 12 months. At both ages, the serum COMP concentration was significantly increased in DMD rats, confirming the diagnostic value of this biomarker (Fig. 7h). In order to assess the translational potential of this novel profibrotic remodelling biomarker in DMD, we immunodetected COMP in muscle sections from DMD patients and controls, confirming the exclusive presence of a high number of COMP-positive cells in the muscle of patients, compared to an almost undetectable level in controls (Fig. 7i–k). COMP expression is therefore an unambiguous biomarker of DMD fibrotic remodelling, with a potential translation value.

## Discussion

In this study we analysed the phenotype of a new DMD preclinical rat model. R-DMDdel52 animals were generated by inducing an out-of-frame deletion in exon 52 of the *Dmd* gene. As a result, no expression of dystrophin was detected in striated muscles, except for rare revertant fibers. Loss of dystrophin was associated with the release of an elevated level of creatine kinase in the bloodstream. This model displayed severe histopathological and progressive aggravated signs of the disease. Indeed, R-DMDdel52 rats showed a progressive deterioration of skeletal muscles with replacement of muscle fibres by noncontractile, connective and adipose tissue, eventually leading to loss of muscle force and mass. The initial reduction in muscle growth, and later in muscle maintenance, could be associated with a reduction in the body mass from the age of 2 months and further prolonged by a marked drop in the body mass curve at 6 months of age, which coincided with fatal deterioration in the general condition of the animals. In R-DMDdel52 rats, CK serum concentration was high in juvenile life, then peaked at 3 months and decreased from 6 months. These results are consistent with the CK levels measured in DMD patients, reported to be the highest at 3–5 years of age followed by a decrease with age and clinical progression [13]. At the histological level, the accumulation and persistence of revertant fibres slightly increased with age, consistent with routine descriptions in human patients, mdx mice and dog models [14, 15, 39, 49]. We also showed a significant loss of type IIb fibres with sparing of type I slow-twitching fibres, similarly to observations in DMD patients and a canine model [67, 71]. This demonstrates that the R-DMDdel52 rat model ranks among those that finely reflect the human condition, characterized by a privileged involvement of glycolytic fibres. Although there is a consensus in the literature on the transition within DMD muscle from fast to slow fibres, an opposite transition has recently been demonstrated in the *extensor carpi ulnaris* muscle of a DMD canine model [21], suggesting that additional analyses on a large panel of muscles are required. The R-DMDdel52 rat model is most appropriate to implement them.

Here we confirm that a preclinical DMD rat model fills a gap between the mildly affected mdx mouse and large animal models, allowing to better characterize at the cellular and molecular levels some insufficiently understood pathogenic mechanisms, as well as to identify those that are still unknown. In addition, our functional analysis focusing on minimally invasive functional parameters, directly comparable to those used in patients, demonstrated that R-DMDdel52 animals provide a highly predictive model of intermediate size, ready for use in preclinical trials. Notably, respiratory capacities of R-DMDdel52 and WT rats by whole-body plethysmography demonstrated functional respiratory defects with a reduced tidal volume at 6 months of age, compensated by and increased respiratory rate. Interestingly, these parameters are significantly impaired in DMD patients starting around 18 years of age [38], and have been associated with diaphragm insufficiency combined with impairment of the abdominal and thoracic accessory skeletal muscles of ventilation, which is exacerbated by scoliosis. In addition to respiratory assessments, we show that grip strength and treadmill walking/running trials yielded reliable, reproducible, and highly significant values that allowed robust discrimination between weaker R-DMDdel52 rats and their healthy WT littermates. The first drop in forelimb muscle strength was detected as soon as 3 weeks of age, confirming an early onset of the dystrophic disease. The assessment of a more integrated motor function was performed using a treadmill test that proved to be also highly discriminant, with performance or endurance values reduced by 50% to 75%, respectively for 3 and 6 months of age. Despite the robustness of the series of values obtained with this test, we chose to no longer use it in routine preclinical evaluation. Indeed, some rats died during the habituation phase of the test, characterized by a very low speed of rotation of the treadmill (2 cm/s). Post-mortem analysis revealed plasma hyperkalaemia, indicating acute rhabdomyolysis with release of intracellular potassium, which likely caused a fatal cardiac arrhythmia. This suggests that any effort, even mild, imposed on DMD rats can be fatal and highlights the extreme spontaneous fragility of these animals. As an alternative to the treadmill test, we are developing quantified observation of the rats filmed during their spontaneous movements. Indeed, DMD rats spontaneously cope very well with the level of exercise they can perform, adjusted to their instant capacities. In other words, they are no longer forced to undertake specific tasks, but cooperate in the collection of relevant biomedical data, in a positive interaction with the investigators.

Cardiac remodelling and failure are main causes of death in Duchenne muscular dystrophy patients [40, 48, 69]. More than 75% of patients with DMD display ECG abnormalities that reveal conduction defects or arrhythmias [41, 62]. Of note, ECG abnormalities are rather easy and inexpensive to characterize, they are otherwise detected at an early age, and they precede the functional decline of the myocardium [30]. They are thus a predictive diagnostic marker for heart failure. They can also be used as a prognostic marker to monitor the capacity of a therapeutic strategy to prevent, slow down or reverse the cardiac remodeling mechanism. Hence, DMD animal models with early presymptomatic ECG outcomes are relevant to assess the efficacy of candidate therapies. Herein, we show that all R-DMDdel52 rats show an abnormal bifid and elongated wave following the QRS, quantifiable via the QTpc parameter that was increased by about 50%. This increased QTpc value is very specific to R-DMDdel52 rats, making QTpc the best functional discriminating criterion, equivalent in reliability to genotyping. Similar modifications, quantified here with neither sedative nor anesthetic molecules known to interfere with ECG values [47], have previously been identified in DMD patients [7, 41, 53, 70]. The prolonged QTpc interval with normal P waves points to a ventricular de- or repolarization defect, likely caused by the progressive cardiac fibrosis observed in DMD rats. Fibrosis is a predictor of adverse outcomes for patients that suffer from cardiomyopathies [23, 27, 54, 68]. On the contrary, a decrease in fibrosis may be a marker of the efficacy of treatments prescribed to restore myocardial contractile function in DMD patients [3]. Further complementary assessment of R-DMDdel52 heart will be useful in the future to more precisely characterize the histological and functional consequences causing, or resulting from, the elevated QTpc. This could include echocardiography, strain rate, and strain analysis using magnetic resonance imaging, as performed in human patients [55].

Although the primary cellular cause of DMD is myonecrosis caused by rupture of the sarcolemma, it becomes more and more obvious that other cell types largely contribute to disease severity and progression, such as macrophages [37, 42, 52, 65] and FAPs [26, 32, 44, 64]. To identify the skeletal muscle cellular and transcriptional modification in DMD, we performed a comparative scRNAseq analysis on diaphragms of R-DMDdel52 and their WT littermates aged 12 months. Adipose tissue and fibrosis deposition within the DMD skeletal muscles are major hallmarks of the pathology, with a prominent role played by FAPs [6, 31, 64]. We thus decided to focus our analysis on this cell population that we found overrepresented in DMD muscles compared to WT. In comparison, such a large increase in cell number has never been observed in the mdx mouse model, in which the FAP number was reported to be only slightly increased [20]. Next, we targeted the scRNAseq analysis by focusing on the transcriptomic changes between WT and R-DMDdel52 samples, and identified COMP as a crucial extracellular matrix protein that could be used as a specific biomarker of muscle fibrosis, as previously done for liver, lung, and skin diseases follow-up [4, 17, 66]. Given that COMP induces collagen secretion and network ECM assembly [22, 57], our data suggests that COMP overexpression could be used as a relevant diagnostic marker of early tissue fibrosis, remodelling, and disease progression. Importantly, we also demonstrated that COMP levels were elevated within the remodelled human DMD muscle, highlighting its translation potential.

To link the remodelling process, including fibrosis, with chronic inflammation that is a known activator, we characterized resident macrophages in skeletal muscles of healthy and R-DMDdel52 rats, and quantified differences in skeletal muscle local inflammation. R-DMDdel52 skeletal muscles contained increased levels of numerous cytokines, with a peak inflammatory phase at 3 months of age that correlates with an increased number of both M1, M2 macrophages, accompanied by a higher number of CD68^−^:CD206^+^ cells. We speculate that CD68^−^:CD206^+^ cells are immature dendritic cells, or macrophages that lost the expression of the surface marker CD68, as recently described in the *utrn*^±^*mdx* DMD mouse model [28]. Interestingly, cytokines levels reached the highest level at 3 months of age, correlating with the peak of both M1 and M2 macrophage presence in DMD muscles. These data support the idea that the peak inflammatory phase in R-DMDdel52 rats is occurring between 3 weeks and 3 months of age with a dampening as disease progresses. Because of the tight correlation between inflammation and fibrosis, linked to poor clinical outcomes for DMD patients [10, 33, 50], further analysis to thoroughly characterize R-DMDdel52 myeloid cells would be relevant. In addition, a thorough analysis of blood vessel morphology, blood flow, vessel biomechanical properties, and 3D microvascular network organization would be required to assess whether capillary density, unchanged in a DMD rat muscle section, has indeed no functional impact on muscle perfusion in DMD condition.

To summarize, we demonstrated that the R-DMDdel52 rat model is a model that provides significant added value in translational medicine. The R-DMDdel52 model reproduces the early severity of the disease on all components of the striated musculature, more faithfully than any other existing rodent model. In this model, we have demonstrated highly discriminating functional, blood, and histological parameters throughout the progression of the disease, thus providing reliable and robust output parameters for evaluating the efficacy of innovative treatments, some of which are already under investigation. Compared to other DMD preclinical animals, this model is part of a preclinical continuum that allows the rat to be used as a first line for validating the most effective strategies among a growing number of proposed candidate compounds. Then, in a second line, the most efficient selected strategies could be tested on a limited number of larger DMD models, which are genetically more heterogeneous and share many environmental factors with humans, making them more suitable to address pharmacokinetics, immune response or precision medicine issues.

## Supplementary Information

**Additional file 1.** **Fig. S1** Myofibre CSA of the diaphragm and morphological evaluation of TA muscle. **Fig. S2** Inflammatory cytokine evaluation and fibre type switching in R-DMDdel52 rats. **Fig. S3** COMP expression in heart and diaphragm

## Data Availability

Data generated or analyzed during this study are included in this published article and its additional files. scRNAseq sequencings have been deposited in the NCBI Gene Expression Omnibus database with the accession code GSE198237.
