# Supplementary material for: Duchenne muscular dystrophy trajectory in R-DMDdel52 preclinical rat model identifies COMP as biomarker of fibrosis
Source: Acta Neuropathol Commun. 2022 Apr 25;10:60. doi: 10.1186/s40478-022-01355-2 (PMC9036715; doi:10.1186/s40478-022-01355-2)
Supplement: Supplementary file 1 — Additional file 1. Fig. S1 Myofibre CSA of the diaphragm and morphological evaluation of TA muscle. Fig. S2 Inflammatory cytokine evaluation and fibre type switching in R-DMDdel52 rats. Fig. S3 COMP expression in heart and diaphragm [file 40478_2022_1355_MOESM1_ESM.pdf]

Acta Neuropathologica Communications
ANEC-D-22-00061

R-DMDdel52 rat model of Duchenne muscular dystrophy for the identification of Comp as biomarker of fibrosis

Valentina Taglietti[1,2,3,6], Kaouthar Kefi[1,2,3], Iwona Bronisz-Budzynka[1,2,3], Busra Mirciloglu[1,2,3], Mathilde Rodrigues[1,2,3], Nastasia Cardone[1,2,3], Fanny Coulpier[1,2,3], Baptiste Periou[1,2,3,4], Christel Gentil[5], Melissa Goddard[1], François-Jérôme Authier[1,2,3,4], France Pietri-Rouxel[5], Edoardo Malfatti[1,2,3,4], Peggy Lafuste[1], Laurent Tiret[1,2,3,6], Frederic Relaix[1,2,3,4,6 *].

1. Univ Paris-Est Créteil, INSERM, U955 IMRB, "Biology of the neuromuscular system" Team, F-94010 Créteil, France
2. Ecole nationale vétérinaire d’Alfort, IMRB, F-94700 Maisons-Alfort, France
3. EFS, IMRB, F-94010 Créteil, France
4. AP-HP, Hôpital Mondor, FHU SENEC, Service de Pathologie, F-94010 Créteil, France
5. Sorbonne Université, INSERM, UMRS974, Center for Research in Myology, F-75013, Paris, France
6. Senior authors

*Correspondence to Frederic Relaix - frederic.relaix@inserm.fr - +33 149 813 940

Supplementary information

Figures S1, S2 and S3.

Figure S1

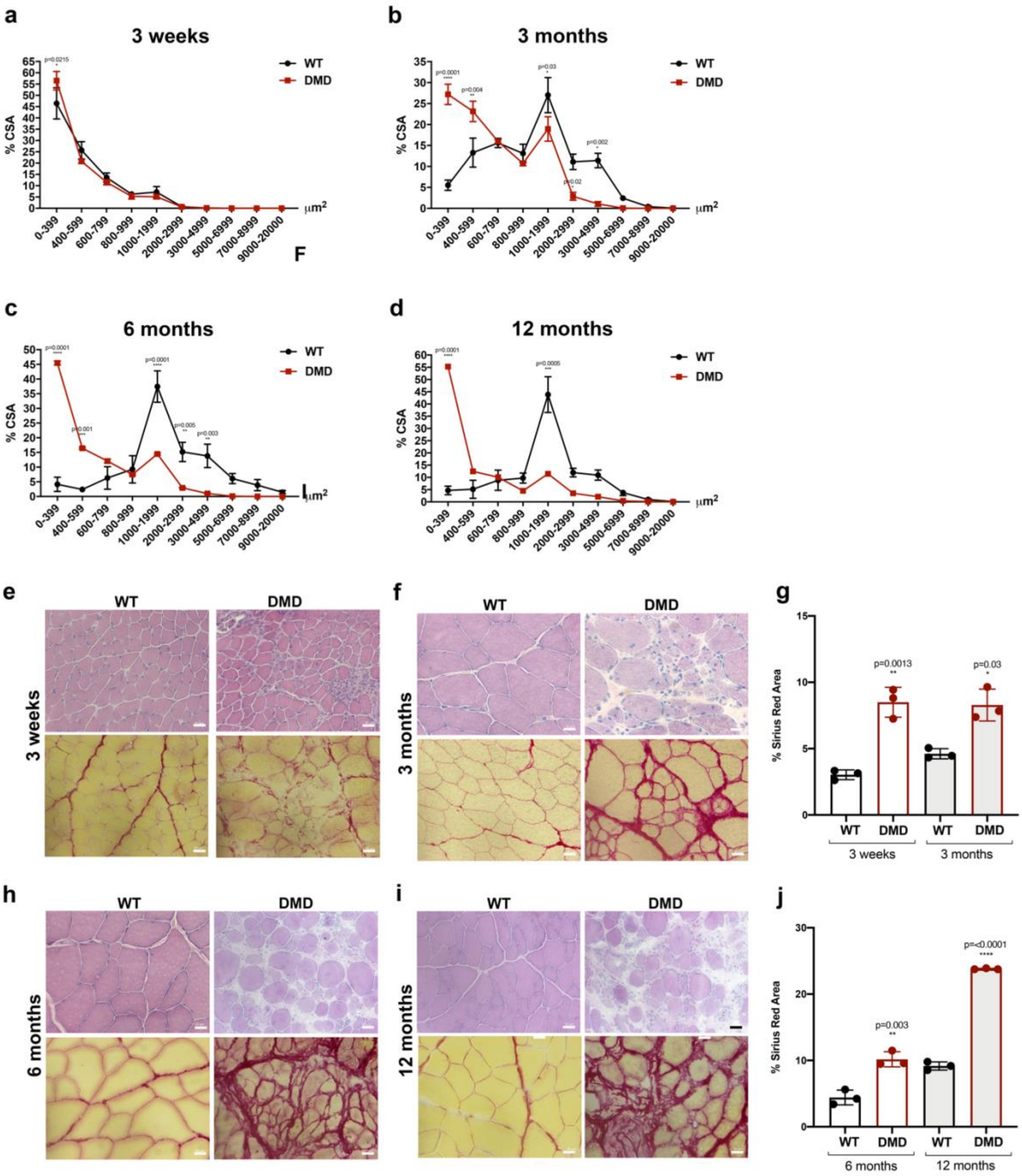

Fig.S1 Myofibre CSA of the diaphragm and morphological evaluation of TA muscle

a) to d) CSA of diaphragm of WT and R-DMDdel52 rats at 3 weeks (a), 3 months (b), 6 months (c) and 12 months of age (d). e) Hematoxylin and eosin (upper panel) and Sirius red (lower panel) staining of *tibialis anterior* of 3-week-old WT and R-DMDdel52 rats (scale bar = 50 μm). f) Hematoxylin and eosin (upper panel) and Sirius red (lower panel) staining of *tibialis anterior* of 3-month-old WT and R-DMDdel52 rats (scale bar = 50 μm). g) Quantification of fibrotic Area of E-F. g) Quantification of fibrotic Area of e-f. h) Hematoxylin and eosin (upper panel) and Sirius red (lower panel) staining of *tibialis anterior* of 6-month-old WT and R-DMDdel52 rats (scale bar = 50 μm). i) Hematoxylin and eosin (upper panel) and Sirius red (lower panel) staining of *tibialis anterior* of 12-month-old WT and R-DMDdel52 rats (scale bar = 50 μm). j) Quantification of fibrotic Area of h-i.

Figure S2

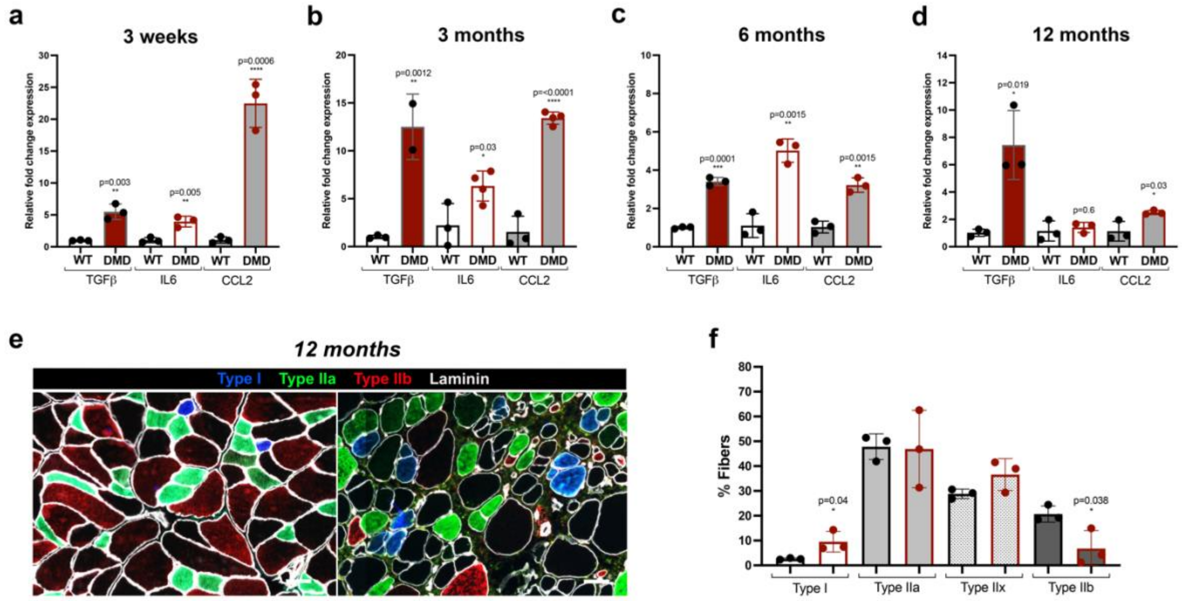

Fig. S2 | Inflammatory cytokine evaluation and fibre type switching in R-DMDdel52 rats.

a-d) qPCR analysis of inflammatory cytokines (*TGFβ, IL6,* and *CCL2*) on quadriceps of WT and R-DMDdel52 rats at 3 weeks (a), 3 months (b), 6 months (c) and 12 months (d). e) Immunofluorescence for Type I (blue), IIa (green), IIx (unstained, black), IIb fibres (red) in WT and R-DMDdel52 EDL at 12 months (scale bar = 50 µm). (f) Quantifications of the percentage of Type I, IIa, IIx, IIb fibres at 12 months of age in WT and R-DMDdel52 EDL muscles. Statistical analysis was performed with unpaired Student's t-test.

Figure S3

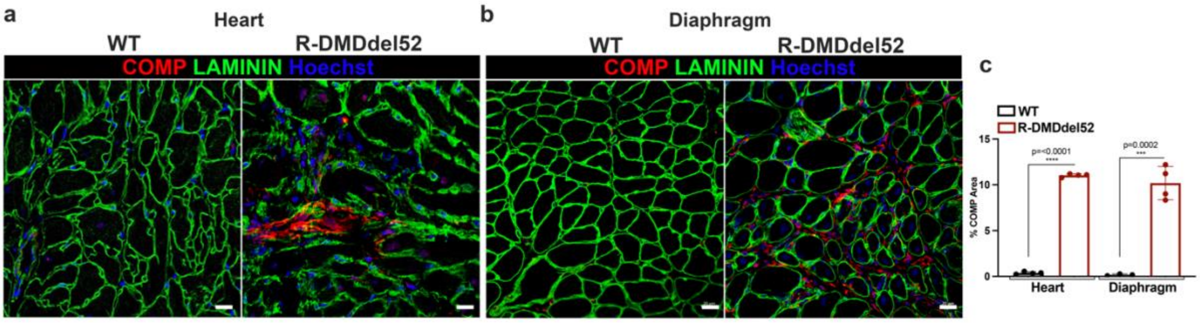

Fig. S3 | COMP expression in heart and diaphragm.

a) and b) Representative immunofluorescences of COMP expression in heart (a) and diaphragm (b) of WT and R-DMDdel52 rat at the age of 12 months. LAMININ is shown in green, and nuclei are stained with Hoechst (scale bar = 50 µm). c) Quantification of COMP positive area in heart and diaphragm at 12 months.
